# Effectiveness of eccentric-biased exercise interventions in reducing the incidence of falls and improving functional performance in older adults: a systematic review

**DOI:** 10.1007/s41999-021-00571-8

**Published:** 2021-10-11

**Authors:** Durga Kulkarni, Sarah Gregory, Michelle Evans

**Affiliations:** 1 Usher Institute, NINE Bioquarter, University of Edinburgh, 9 Little France Road, Edinburgh, EH16 4UX Scotland, UK; 2 Outpatient Department 2, Edinburgh Dementia Prevention, Western General Hospital, Edinburgh, EH4 2XU UK

**Keywords:** Eccentric exercise, Functional, Randomised-controlled trials, Elderly, Systematic review

## Abstract

**Background:**

Exercise has been known to preserve and enhance functional performance in older adults. Eccentric exercise involves muscle contractions characterised by unique features such as lengthening of the muscle–tendon complex by a greater opposing force.

**Aims:**

To systematically review randomised-controlled trials (RCTs) investigating the effectiveness of eccentric exercises in reducing the incidence of falls and improving the functional performance in older adults.

**Key methods:**

We conducted a systematic review of RCTs following the PRISMA-P guidelines. Searches were completed in the Cochrane Central Register of Controlled Trials, Embase, CINAHL, Medline, and Global Health CABI. We included RCTs reporting at least one of the following outcomes—falls, Berg balance scale, timed-up and go test, chair stand test, stair climb test, maximal walking speed, and minute walking distance. Two reviewers screened papers for eligibility and assessed the quality of included papers using the Cochrane Collaborative risk-of-bias tool for randomised trials. Data were extracted by a single reviewer and cross-checked by the second reviewer. A narrative synthesis was undertaken, given the high level of heterogeneity across studies.

**Results:**

Ten studies were assessed as eligible for inclusion in the review. Overall, eccentric exercises were as effective as conventional resistance exercises in improving the selected outcomes by most studies. Additionally, when pre-exercise and post-eccentric exercise functional performance measures were compared, there was a statistically significant improvement in nearly all measures. The quality of trials was mixed (one high, four moderate, two low–moderate, and three low risk of bias).

**Conclusions:**

Our systematic review suggests that eccentric exercises can be as effective as conventional exercises in older adults. Evidence suggests that such exercise interventions can lead to an improvement in geriatric function.

**Supplementary Information:**

The online version contains supplementary material available at 10.1007/s41999-021-00571-8.

## Key summary points

**Aim:**

To systematically review the literature on the effectiveness of eccentric exercise interventions in reducing falls and improving the functional performance in older adults.

**Findings:**

The existing literature was of mixed quality and suggested that eccentric exercises can be as effective as conventional exercises in improving functional performance in healthy older adults. There was limited evidence focussing on the aspect of incidence of falls.

**Message:**

Eccentric exercises may be as effective as conventional exercises in improving geriatric function, although evidence remains limited. More research is needed to explore any adverse effects of such exercises in older adults.

**Supplementary Information:**

The online version contains supplementary material available at 10.1007/s41999-021-00571-8.

## Introduction

Functional dependence in activities of daily living (ADLs) and falls are significant challenges for a globally ageing population [1]. It is estimated that 28–35% of people aged > 65 years fall each year increasing to 32–42% for those aged > 70 years [2]. Balance problems are common in the older adult population that can often lead to falls [3]. Falls and the inability to perform ADLs impede a person from meeting the WHO definition of healthy ageing [4]. A key part of the WHO definition of healthy ageing is maintaining a functional and cognitive ability, which includes the ability to meet basic needs, learn, grow and make decisions, remain mobile, build and maintain relationships, and contribute to society [4]. Some non-fatal outcomes of falls like bone fractures, depression, immobilisation, social isolation, and protective attitudes can result in reduced functional independence in the older adult population and challenge the healthy ageing process [5, 6]. Some of these outcomes in turn act as risk factors for repeated falls in the future [7]. This establishes a vicious cycle that aggravates the decline in the quality of life (QOL) of older adults with far-reaching physical and psychological implications on the older adult population and their caregivers and families. From an economic perspective, such events incur considerable dependency costs to the families and the wider society [8].

Exercise is one of the preventative strategies that can be applied at the individual level. Exercise has multiple benefits in the older adult population, including improvement in physical strength and muscular coordination and control [9]. These effects of physical exercise are known to contribute to improved fall prevention strategies and improvement in functional performance. However, it is important to bear in mind that different levels of decline in physiological function and cardio-respiratory capacities occur as part of the physiological ageing process depending on the activity levels of individuals [10]. Older adults exhibit a greater reduction in exercise tolerance compared to the young and the effects of de-training after cessation of exercise are more pronounced in older adults than their younger counterparts [11]. Thus, safety and viability must form vital components of exercise prescription in this population.

Eccentric muscle contraction or eccentric control is a process in which a muscle actively develops force, but is lengthened by a greater opposing force and is characterised by high force production [12, 13]. The three important functions performed by eccentric muscle contractions are deceleration of a limb or limb part, force absorption, and controlling a movement against gravity/an external force. Most of the lower limb muscles must function in a controlled manner against gravity or support the bodyweight to maintain an upright position against gravity in day-to-day activities, and hence, eccentric muscle contractions are intrinsic to many daily activities [14]. Eccentric exercise interventions involve or focus on eccentric contractions and can be delivered using a wide array of exercises, equipment, and techniques. A few examples include fixing or stabilising a limb in a specific position against gravity or any other force (like an elastic force of resistance bands); steady movement in the direction of external force and downhill walking or stair descent [15].

Minimising or delaying the anatomical and physiological impairments secondary to the ageing process can support older adults to practice employment, volunteering, household, and self-care activities effectively, and maintain a good QOL. Older adults’ fitness has become more pertinent after the challenges faced by the aged and ageing during the COVID-19 pandemic [16]. This situation further highlights the importance of finding safe, feasible, and effective exercise regimens that can be undertaken by this population [17].

Pure eccentric exercise is unachievable without any specialised equipment or external assistance that allows the elimination of the active concentric phase. Our study is, therefore, centred on eccentric-biased exercises, i.e., exercises that focus on the eccentric phase, but do not eliminate the concentric phase. For this review, eccentric exercise should be interpreted as eccentric-biased exercises hereafter. This review considers a few of the functional outcome measures, which are commonly measured as outcomes and relevant in older adults.

This study aims to critically analyse and systematically review existing relevant literature to determine the effectiveness and safety of eccentric exercise in improving functional performance in the older adult population to allow evidence-based physical therapy practice and decision-making.

## Aims and objectives

### Aims

To identify, appraise, and analyse randomised-controlled trials (RCTs) investigating the effectiveness of eccentric muscle strengthening in reducing the incidence of falls and improving functional performance in the older adult population to help inform future older adults’ care practices.

### Objectives

1. To critically assess the effectiveness of eccentric muscular exercise in improving balance and lowering the incidence of falls in older adults.
2. To critically assess the effect of eccentric muscle strengthening on functional performance in older adults.
3. To critically assess the incidence of injuries or episodes of soreness secondary to participation in eccentric muscle strengthening programmes in older adults

### Methods

This systematic review was guided by the Preferred Reporting Items for Systematic Review and Meta-Analysis Protocols (PRISMA-P 2015) guidelines [18]. PRISMA-P 2015 was utilised for the preparation and reporting of a protocol for this systematic review to help direct the review process. The study protocol was published on PROSPERO (CRD42020211896). For this review, older adults were defined as those aged ≥ 60 years, in line with the commonly used criteria by the World Health Organisation (WHO) to refer to the ageing population [19].

We developed a comprehensive search strategy and ran this in five health-related databases, namely the Cochrane Central Register of Controlled Trials, Embase, CINAHL, Medline, and Global Health CABI. The searches were reviewed by a specialist academic medical librarian. Additionally, we practiced citation tracking of included studies to identify any potentially relevant content. Initial searches were run on 7th August 2020 with an update search run on 18th December 2020. The searches were not limited to any specific timeframe or geographical locations. The selection of trials was restricted to those published in the English language due to the unfamiliarity of the reviewer team with other languages.

The studies had to meet the following criteria:

1. Population: healthy participants aged ≥  60 years.
2. Intervention: eccentric strengthening or eccentric-biased strengthening intervention.
3. Comparator: control (no exercise intervention) or concentric exercise.
4. Outcome/s: trials assessing at least one of the following- incidence of falls, Berg balance scale (BBS) measure, timed-up and go (TUG) score, maximal walking speed (MWS), stair climb test (SCT), minute walking distance (MWD), and chair stand time (CST). Rate of perceived exertion and incidence of muscle soreness were secondary outcomes of this review. We included studies that reported at least one of our primary outcomes. Studies were still included if they did not report the secondary outcomes.
5. Study design: randomised control trials (RCTs).

The inclusion and exclusion criteria are outlined in Table 1.

**Table 1:** Eligibility criteria for inclusion of studies in the review

	Inclusion	Exclusion
Population	Adults aged 60 years and above	Studies recruiting older adults who were professional or elite athletes Studies recruiting older adults diagnosed with any neurological, musculoskeletal, cardio-respiratory condition, or cognitive impairment
Intervention	Pure eccentric strengthening or combined eccentric-biased strengthening intervention	Eccentric–concentric strengthening interventions with equal components of eccentric and concentric muscle contractions
Comparator	Control Concentric training	No comparator group
Outcomes	Primary outcomes Studies reporting at least one of the following primary outcomes will be included: 1. Incidence of falls 2. BBS measure 3. TUG score 4. MWS 5. SCT 6. MWD 7. CST Secondary outcomes^a^ 1. Muscle soreness after exercise or incidence of injuries 2. Rate of perceived exertion	Studies not reporting at least one of the primary outcomes
Study design	Randomised-controlled trials (RCTs)	Observational studies (case studies, case reports, cross-sectional studies, ecological studies, cross-sectional studies, and cohort studies) Qualitative research Modelling studies Narrative reviews Systematic reviews Quasi-randomised, cross-over, and single-arm trials

^a^RCTs were included if they reported at least one primary outcome and regardless of secondary outcomes. Data for secondary outcomes were extracted if available.

The search terms are used in each database and the justification behind the exclusion of studies at the full-text screening stage is provided in the appendix. Deduplication of the search results was performed in EndNote X9. Two reviewers (DK and SG) independently conducted the title and abstract screening and the full-text screening for study selection based on these pre-determined criteria. DK developed and piloted the data extraction form and used the finalised form in the MS Excel to complete data extraction. SG performed a check of the data extraction to ensure all required data had been accurately extracted from the studies.

DK and SG independently assessed the methodological risk of bias of each study by employing version 2 of the Cochrane risk-of-bias tool for RCTs (RoB 2) [20].

Any disagreement between the two reviewers at any step was resolved by discussion between DK, SG, and ME.

In the case of trials reporting multiple follow-up points, data were extracted for that follow-up which had been stated in the hypothesis and that was mentioned in the primary objectives of the trials. Similarly, for trials involving eccentric, concentric, and control groups, data were extracted for the concentric and eccentric groups only as they allowed a direct comparison between two exercise interventions and allowed accounting for the social aspects of training.

A trial by Hill et al. fits the pre-determined eligibility criteria [21]. The findings of the paper suggested that eccentric interventions increase the risk of falls and degrade functional performance in older adults. This was understandable given that the outcomes were measured extremely short term, i.e., within several minutes after the administration of a single session of eccentric exercise. Due to a gross difference in the intervention delivery (single session) between this trial and other trials included in the review, this study by Hill et al. was excluded from the following narrative synthesis. We finally included ten studies in our analyses.

Included studies varied remarkably in terms of the methods of delivering the intervention, intervention delivered to the comparator group, exercise intensities, frequency of training, rate of progression of exercise intensity, length of follow-up, and outcomes measured. Therefore, narrative analysis was chosen to be the method of analysis, and a meta-analysis was ruled out. Because of the inability to pool the results, assessment for publication bias was not possible.

**Fig. 1:**
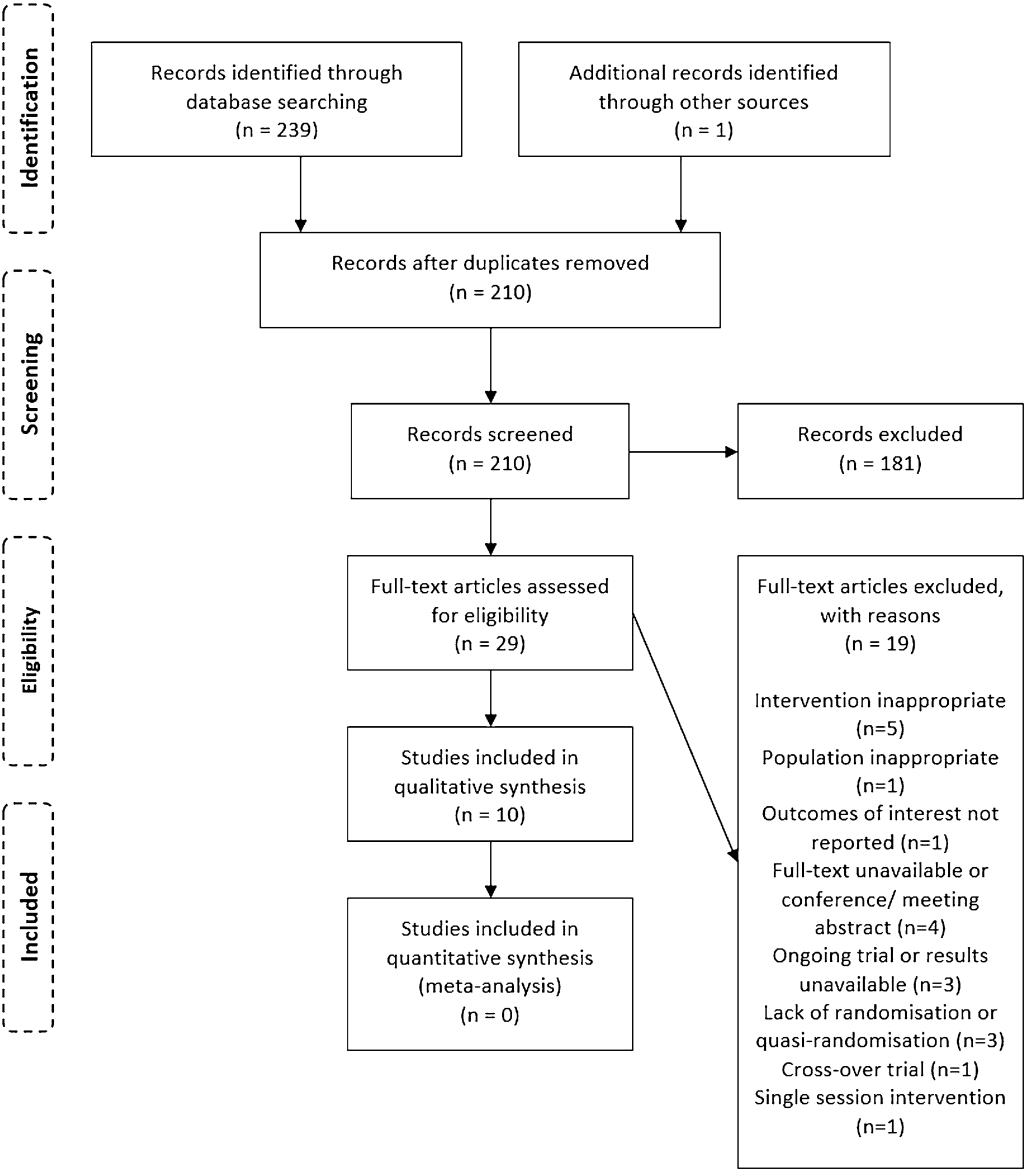
PRISMA diagram

The PRISMA diagram (Fig. 1) illustrates the flow of studies at each stage.

## Results

### Study characteristics

The characteristics of included studies have been summarised in Table 2.

**Table 2:** Table summarising the characteristics of the studies included in the systematic review

Author, year of publication	Geographical location	Age of participants in years (mean ± S.D. and/ or range)	Length of follow-up	Sample size (Total sample size = eccentric intervention + comparator + any other intervention)	Progression of exercise intensity in the intervention group	Training frequency of the intervention group	Intervention	Comparator	Dropouts (Total dropouts = intervention dropouts + comparator dropouts)
Dias et al. (2015) [22]	Brazil	67 ± 6	12 weeks	26 = 13 + 13	Every 2 weeks based on pre-training RM	2x/week	Training with 1.5 s concentric phase and 4.5 s eccentric phase	Training with 1.5 s concentric phase and 1.5 s eccentric phase	7 = 4 + 3
Gault et al. (2012) [23]	United Kingdom	67 ± 4	12 weeks	24 = 13 + 11	Every 4 weeks based on self-selected walking speed (perceived by participants to be able to maintain for 30 min)	1x/week	Downslope treadmill walking (treadmill gradient − 10%)	Level treadmill walking (treadmill gradient 0%)	6 = 3 + 3
Johnson et al. (2019) [24]	United States	68.2 ± 3.7	8 weeks	30 = 15 + 15	Every week or after every 3 weeks based on RPE	2x/week	Training on eccentric step machine	Control group	1 = 1 + 0
Katsura et al. (2019) [25]	Japan	71.6 ± 5.6	8 weeks	22	After about every 2 weeks based on RPE	1x/week at the training centre and ≥ 2x/week at home	Eccentric basic manual resistance exercises without equipment	Concentric basic manual resistance exercises without equipment	5
LaStayo et al. (2017) [26]	United States	76.1 (range 65–93)	1 year	134 = 68 + 66	After 2 weeks initially and weekly thereafter based on Borg’s rating	3x/week	Training using recumbent step ergometer	Traditional training	22 = 14 + 8
Mueller et al. (2009) [27]	Switzerland	80.6 (range 71–89)	12 weeks	62 = 23 + 23 + 16	Load increased in 5-min steps until 20 min for the first few sessions. Thereafter, load ramped every session by 20% of the initial maximal power output	2x/week	Training on recumbent ergometer	Conventional resistance training	13 = 3 + 6 + 4
Raj et al. (2012) [28]	Australia	68 ± 5	16 weeks	28 = 13 + 12 + 3	Every after 2 weeks and every 3 weeks thereafter based on RM	2x/week	Concentric lifts bilaterally with 50% of 1 RM and eccentric lowering unilaterally	Training completely bilateral interventions at 75% of 1 RM	0
Sañudo et al. (2019) [29]	Spain	65 ± 4	6 weeks	36 = 18 + 18	Unclear	2x/week or 3x/week for alternate weeks	Flywheel resistance exercise training	Control group	2 = 1 + 1
Sanudo et al. (2020) [14]	Spain	64 ± 5 (range 57–75)	6 weeks	36 = 18 + 18	Every week based on moment inertia	2x/week or 3x/week for alternate weeks	Flywheel resistance exercise training	Control group	0
Symons et al. (2005) [30]	Canada	range 65- 87	12 weeks	37 = 14 + 10 + 13	Every session based on highest peak torque value	3x/week	Maximal eccentric contractions	Maximal concentric contractions	5 = 5 + 0 + 0

### Study findings: primary outcomes

There was significant heterogeneity in the number and types of outcomes reported by trials. We also observed a substantial diversity in the measurement of a particular outcome across trials. The details regarding the exact procedure adopted by different studies to measure these outcomes are attached in the appendix.

### Analysis of comparisons between the pre-intervention and post-intervention measures

The findings of comparisons between baseline and post-eccentric exercise intervention measures are summarised in Table 3.

**Table 3:** Study findings—analysis of the effectiveness of eccentric interventions—comparison of pre- eccentric intervention and post-eccentric intervention measures

Outcome	Study	Effect magnitude	Effect direction	Statistical significance
BBS	Johnson et al. [24]	Not specified	Increased, i.e., improved performance	*p* = 0.014*
	Mueller et al. [27]	*P*ercent change ± S.D. = 1.7 ± 0.3%	Increased, i.e., improved performance	*p* > 0.05
TUG	Dias et al. [22]	Not specified	Reduced, i.e., improved performance	*p* < 0.001***
	Gault et al. [23]	− 22% change in time	Reduced, i.e., improved performance	*p* < 0.01**
	Johnson et al. [24]	Not specified	Reduced, i.e., improved performance	*p* = 0.001**
	Katsura et al. [25]	Percent change ± S.D.in time = − 16.7 ± 9.9%	Reduced, i.e., improved performance	*p* = 0.001**
	Mueller et al. [27]	Percent change ± S.D. = − 7.5 ± 0.2%	Reduced, i.e., improved performance	*p* < 0.05*
	Sanudo et al. (2019) [29]	Change (mean ± S.D.) from 6.25 ± 1.38 s to 5.42 ± 0.74 s	Reduced, i.e., improved performance	*p* < 0.01**
	Raj et al. [28]	Not specified	Reduced, i.e., improved performance	*p* = 0.08
SCT	Dias et al. [22]	Not specified	Reduced, i.e., improved performance	*p* < 0.001***
	Symons et al. [30]	Not specified	Reduced, i.e., improved performance	*p* < 0.03*
MWS	Dias et al. [22]	Not specified	Reduced, i.e., improved performance	*p* = 0.004**
	Gault et al. [23]	Percent change ± S.D. in s*p*eed = 22 ± 11%	Increased, i.e., improved performance	*p* < 0.01**
	Sanudo et al. (2020) [14]	Change (mean ± S.D.) from 4.89 ± 3.07 m/s to 4.66 ± 0.60 m/s	Reduced, i.e., worsened performance	*p* = 0.018*
	Raj et al. [28]	Change (mean ± S.D.) from 2.79 ± 0.32 to 2.60 ± 0.29	Reduced, i.e., improved performance	*p* < 0.01**
MWD	LaStayo et al. [26]	Change (mean (95% C.I.)) in distance from 405.21 m (367.40, 443.03) to 439.18 m (394.65, 487.72)	Increased, i.e., improved performance	Unclear
CST	Dias et al. [22]	Not specified	Reduced, i.e., improved performance	*p* < 0.001***
	Gault et al. [23]	Percent change ± S.D. in time = -34 ± 8%	Reduced, i.e., improved performance	*p* < 0.01**
	Johnson et al. [24]	Not specified	Increased, i.e., improved performance	*p* = 0.001**
	Katsura et al. [25]	Not specified	Increased, i.e., improved performance	*p* < 0.01**
	Sanudo et al. (2020) [14]	Change (mean ± S.D.) from 12.67 ± 3.07 to 14.94 ± 2.80	Increased, i.e., improved performance	*p* < 0.001***

*CI* confidence interval, *SD* standard deviation, *m* metre, *m/s* metres per second

*Statistically significant at 5% significance level

**Statistically significant at 1% significance level

***Statistically significant at 0.1% significance level

### Analysis of comparisons between the eccentric intervention groups and the comparator groups

The findings for comparisons between the effectiveness of eccentric interventions and either concentric intervention or control (i.e., no intervention) are summarised in Table 4.

**Table 4:** Study findings—analysis of the effectiveness of eccentric interventions—comparison of functional outcome measures in the intervention and comparator groups

Outcome	Study	Effect magnitude	Effect direction	Statistical significance
BBS	*Johnson* *et* *al.* [24]	*Not* specified	*Greater* *improvement* *in* *the* *intervention* *group*	p = 0.003**
	**Mueller** **et** **al*.*** [27]	**Scores** **improved** **in** **both** **groups.** **Reduction** **in** **time** **(mean** **percent ± S.D.)** **in** **the** **intervention** **group = 1.7 ± 0.2%** **and** **the** **comparator** **group = 0.7 ± 0.3%**	**Greater** **improvement** **in** **the** **intervention** **group**	***p* > 0.05**
TUG	**Dias** **et** **al.** [22]	**(change ± S.D.)** **in** **the** **intervention** **group = (–** **15.89 ± 8.82)** **and** **the** **comparator** **group = (–** **11.02 ± 4.60)**	**Greater** **improvement** **in** **the** **intervention** **group**	***p* = 0.165**
	**Gault** **et** **al*.*** [23]	**not** **specified** **(time** **improved** **in** **both** **groups)**	**Greater** **improvement** **in** **the** **intervention** **group**	***p* > 0.05**
	*Johnson* *et* *al.* [24]	*Not* *specified*	*Greater* *improvement* *in* *the* *intervention* *group*	p < 0.001***
	**Katsura** **et** **al*.*** [25]	**Time** **reduced** **in** **both** **groups** **(not** **specified)**	**Greater** **improvement** **in** **the** **intervention** **group**	*p* = 0.045*
	**Mueller** **et** **al*.*** [27]	**Time** **reduced** **in** **both** **groups;** **reduction** **in** **time** **(mean** **percent ± S.D.)** **in** **the** **intervention** **group = –** **7.5 ± 0.2%** **and** **in** **the** **comparator** **group = –** **7.3 ± 0.2%**	**Greater** **improvement** **in** **the** **intervention** **group**	***p* > 0.05**
	*Sanudo* *et* *al.* *(2019)* [29]	*difference* *in* *post–* *intervention* *change* *between* *the* *intervention* *group* *and* *the* *comparator* *group* *(change* *(95%* *CI)) = –* *68* *(–* *1.25* *to* *–* *98)*	*Greater* *improvement* *in* *the* *intervention* *group*	p = 0.023*
	**Raj** **et** **al*.*** [28]	**Not** **reported**	**Not** **reported**	**Not** **re*p*orted**
CST	**Dias** **et** **al*.*** [22]	**(percent** **changes ± S.D.)** **change** **in** **the** **intervention** **group = (–** **15.89 ± 8.82%)** **and** **conventional** **training** **group** **(–** **11.02 ± 4.60%)**	**Greater** **improvement** **in** **the** **intervention** **group**	***p* = 0.349**
	**Symons** **et** **al*.*** [30]	**Not** **specified** **(step** **time** **reduced** **in** **both** **groups)**	**Unclear**	***p* > 0.05**
MWS	**Dias** **et** **al*.*** [22]	**(percent** **changes ± S.D)** **in** **the** **intervention** **group** **(–** **11.83 ± 9.40%)** **and** **the** **comparator** **group = (–** **8.54 ± 10.65%)**	**Greater** **improvement** **in** **the** **intervention** **group**	***p* = 0.484**
	**Gault** **et** **al*.*** [23]	**not** **Specified** **(speed** **improved** **in** **both** **groups)**	**Greater** **improvement** **in** **the** **intervention** **group**	***p* > 0.05**
	*Sanudo* *et* *al.* *(2020)* [14]	*Difference* *in* *post–* *intervention* *change* *between* *the* *intervention* *group* *and* *the* *comparator* *group* *(change* *(95%* *CI)) = –* *0.20* *(–* *0.44* *to* *0.04)* *m/s*	*Greater* *worsening* *of* *performance* *in* *the* *intervention* *group*	*p = 0.095*
	**Raj** **et** **al*.*** [28]	**not** **Reported**	**not** **Reported**	**not** **reported**
MWD	**LaStayo** **et** **al*.*** [26]	**Mean** **change** **in** **intervention** **group** **was** **33.97** **and** **the** **comparator** **group** **was** **23.05**	**Greater** **improvement** **in** **the** **intervention** **group**	***p* = 0.565**
CST	**Dias** **et** **al*.*** [22]	**(change ± S.D.)** **in** **the** **intervention** **group = (–** **15.02 ± 5.95%)** **and** **the** **comparator** **group = (–** **15.99 ± 7.47%)**	**Lesser** **improvement** **in** **the** **intervention** **group**	***p* = 0.756**
	**Gault** **et** **al*.*** [23]	**not** **specified** **(time** **improved** **in** **both** **groups)**	**Unclear**	***p* > 0.05**
	Johnson et al. [24]	not Specified (number of repetitions increased in both groups)	Greater improvement in the intervention group	*p* = 0.07
	**Katsura** **et** **al*.*** [25]	**Increase** **in** **number** **of** **repetitions** **in** **both** **groups.** **p = 0.049**	**Greater** **improvement** **in** **the** **intervention** **group**	*p* = 0.049*
	*Sanudo* *et* *al.* *(2020)* [14]	*difference* *in* *post–* *intervention* *change* *between* *the* *intervention* *group* *and* *the* *comparator* *group* *(change* *(95%* *CI)) = 1.67* *(0.53* *to* *2.80)* *m/s*	*Greater* *improvement* *in* *the* *intervention* *group*	p = 0.005**
Falls	**LaStayo** **et** **al*.*** [26]	**Number** **of** **days** **survived** **without** **a** **fall** **(mean** **days ± S.D.)** **in** **the** **intervention** **group = 239.00 ± 18.00** **and** **the** **comparator** **group = 249.67 ± 16.38**	**Greater** **improvement** **in** **the** **comparator** **group**	***p* = 0.565**

*CI:* confidence interval, *SD* standard deviation

*Statistically significant at 5% significance level

**Statistically significant at 1% significance level

***Statistically significant at 0.1% significance level

The boldicized cell implies that the comparator group was trained with a concentric training protocol, and colourless or italicized cell indicates that the comparator group was not under training, i.e., a control group

### Study findings: secondary outcomes

Only two studies analysed the secondary outcomes of interest. The mean rate of perceived exertion was consistently higher in the conventional training group than in the intervention group in the Raj et al. trial [28]. Katsura et al. observed that the mean peak muscle soreness indicated by VAS recorded at 1 or 2 days after each session, for the eight sessions, was slightly higher in the intervention group (10.0 ± 3.0 mm) than the comparator group (14.0 ± 4.0 mm). However, the mean difference between the two groups was statistically insignificant [25].

### Risk of bias assessment

The summary of the risk of bias assessment was conducted using the Cochrane risk of bias assessment tools. Studies were graded based on bias arising from the randomisation process; bias due to deviations from intended interventions (including the effect of assignment to and adherence to interventions); missing outcome data; risk of bias in the measurement of outcomes; risk of bias in the selection of the reported outcome. Every study was rated on each of these six domains as low, moderate, or high risk of bias and an overall risk of bias score was calculated.

**Fig. 2:**
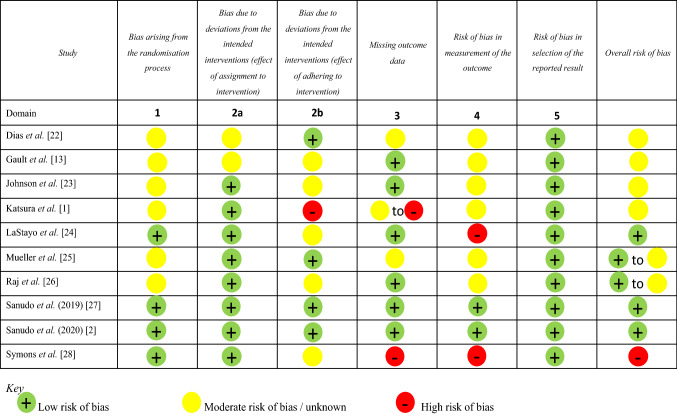
Risk of bias assessment of individual studies using the Cochrane collaboration tool for RCTs (created using Microsoft PowerPoint)

Figure 2 represents the risk of bias assessment results.

**Fig. 3:**
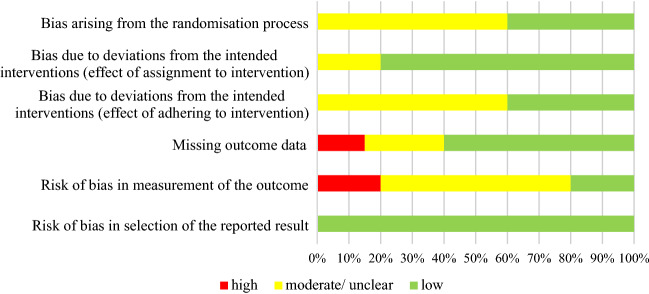
Risk of bias graph: review authors' judgements about each risk of bias item presented as percentages across included studies (*n* = 10)—created using Microsoft Excel

Figure 3 shows the review authors’ judgements about each risk of bias item for the review presented as percentages across included studies.

Based on principles from the Grading of Recommendations Assessment, Development, and Evaluation (GRADE), the quality of evidence generated by this systematic review was assessed for each outcome separately [31]. For submaximal outcomes like 6MWD [26] and self-paced SCT [30], the quality of evidence was regarded as very low due to the effect of non-blinding of assessors that can influence the degree of motivation provided to the participants. Second, these precise outcomes were reported by only one study (although SCT was measured by two trials [22, 30], self-paced SCT was measured by a single trial) each, and therefore, further research is required to build robust evidence. For other outcomes, the quality of evidence is regarded as low (a systematic review based on primary studies of predominant moderate quality).

## Discussion

This systematic review was undertaken to explore the effectiveness of eccentric-biased exercises in reducing falls and improving functional outcomes to help guide future older adults’ care. The findings of our review suggest that although there is a lack of consensus among studies regarding the effectiveness of eccentric exercise compared to concentric exercise, findings are consistent concerning the functional improvement post-eccentric exercise intervention compared to the pre-intervention performance. An improvement in these functional outcome measures in the older adult population suggests an improvement in function and is thus likely to contribute towards enabling healthy ageing as defined by the WHO [4].

Except for BBS in Mueller et al. and TUG in Raj et al., all studies showed statistically significant improvement in the functional outcomes after eccentric exercise intervention compared to the baseline measures (Table 3) [27, 28]. The participants recruited in the Mueller et al.’s study were higher functioning older adults and characterised by an active lifestyle with concomitant duties and obligations. Functional tests like BBS are less sensitive in such individuals and exhibit a ceiling effect [32]. This implies that the BBS tasks were possibly not challenging enough to detect problems and, eventually, any improvements in the study participants. Although TUG did not give statistically significant results in Raj et al., the findings of this study approached statistical significance (*p* = 0.08). It also seems unlikely that a trial of this size would have the power to detect a change, and thus, the trial approaching significance did not come as a surprise. More conclusive statements about this study could be made after replication of the study with a larger sample.

Data on the incidence of falls were limited. Only one study [26] compared the days survived without a fall in participants performing eccentric exercises and concentric exercises. Older adults performing concentric exercises were observed to survive greater number of days than those performing eccentric exercises. However, the difference was statistically insignificant. It is important to highlight that the precision of measuring the number of fall or near fall events was likely to be low due its subjective nature. Furthermore, it remains to be seen if the incidence of falls after eccentric exercise is lower than that without any type of exercise (control group).

Overall, the results of this systematic review suggested that eccentric exercise interventions are as effective as concentric interventions in improving functional outcomes in older adults. All [22, 23, 26, 27, 30] but one [25] study showed statistically insignificant differences in improvement of functional outcomes after concentric or conventional exercises versus eccentric exercises. The trial by Katsura et al. reported greater improvement in functional outcomes after eccentric exercise compared to concentric exercise. However, this trial was identified as being at a high risk of bias due to deviations from assigned intervention as at least two sessions per week were unsupervised during the study period. The only study that reported days survived without a fall, observed a statistically insignificant difference, such that the concentric exercise group survived greater days without a fall than the eccentric exercise [26]. However, this trial involved 3 months of supervised training, and outcomes were assessed after 1 year. Therefore, inconsistency in exercise levels and adherence to protocol over the year might have influenced the findings. Additionally, evidence regarding the duration for which eccentric training effects are endured, the minimum frequency required to endure eccentric training effects in the long term, and effects of eccentric de-training or reversal of training after cessation of exercise in the older adult population do not seem to currently exist.

Trials that compared the effect of eccentric exercises to control or no intervention group showed a statistically significant difference between the improvement in the two groups for all outcomes except maximal walking speed in Sanudo et al. (2020) and CST in Johnson et al. [14, 24]. It is important to note that the CST in the Johnson et al.’s trial approached statistical significance (*p* = 0.07).

Eccentric exercise interventions can be practised at home or outdoors without any specialised equipment. Techniques like stair-climbing or downhill walking can be undertaken in most places without any financial investment. Katsura et al. demonstrated in their trial that eccentric basic manual exercises without any equipment were effective in improving functional measures in older adult participants [25]. These exercises involved focussing on the eccentric phase during activities like chair squat, push squat, calf raise and down, push-ups, rowing, etc. Similarly, eccentric training focussed protocols can be practised by maximising the eccentric phase and minimising the concentric phase during weight training as in the intervention in Dias et al. study [22]. Readily available materials like sand, leftover water bottles or milk cans, hardware materials, rice grains, and cloth rags, etc. can be innovatively utilised to serve as excellent and low-cost replacements for specially designed exercise weights. Bodyweight may be considered as the best resistance particularly in the initial phases of exercise regimens in older adults and when the aim of the exercise is an improvement in function [33]. Thus, we can say that despite the unique nature and physiology behind this exercise, it does not demand unique or specialised equipment and significant investment.

Raj et al. observed that the mean rate of perceived exertion was consistently higher in the conventional training group than in the intervention group [28]. Although Katsura et al. reported a higher mean peak muscle soreness in the intervention group compared to the conventional training group, the difference was not significant [25]. Except these two trials, no other included trials reported any analyses of secondary outcomes of the review. Although none of the studies formally analysed the findings for the secondary outcomes, it may be assumed that the trials did not observe any major event of injury or undesirable outcomes after the intervention. This seems reasonable given that all the trials recruited gradual progression of exercise intensity. However, the studies included in this review predominantly recruited healthy older adults. Therefore, the safety factor needs to be interpreted with a caveat and these findings may not be directly transferred to the frailer or clinical groups as they are likely to have a dissimilar safety profile. Such groups may be at a greater risk of exercise-related injuries like episodes of severe muscle soreness.

Except for a study conducted in Brazil [22], the remaining nine trials were conducted in high-income countries. Apart from economic feasibility, evaluating the acceptability of such interventions in different cultural settings remains essential.

Prescription of eccentric exercises in older adults as opposed to conventional exercises may have a benefit in terms of reduced metabolic cost [12, 34]. This, if true, will be more relevant in the older adult population compared to the general adult population. This means that individuals with limited exercise capacity or limited baseline exercise levels and those with comorbidities may be able to undertake this type of exercise safely. However, it must be noted that despite lower metabolic costs, such eccentric exercises are characterised by high force production and any substantial change in the normal pattern of muscle use (including changes in nature and magnitude of force) can result in muscle damage [12]. Thus, to avoid such muscle damage, intensity should be scaled up at a gentle pace to allow the older adult exercisers to get accustomed to the activity and avoid any untoward incidences of injuries. Additionally, baseline exercise levels of individuals must be considered while planning exercise protocols. Supervised sessions at least in the initial phases of training for beginners might be worthwhile again to avoid any incidences of falls and injuries, and to overcome any fear and/or concern associated with this unfamiliar exercise.

Based on evidence emerging from included studies, a duration as short as about 6–12 weeks of regular eccentric exercise seems sufficient to illustrate its beneficial effects.

Our study has several strengths. We followed the PRISMA-P guidelines and used validated tools. Important decisions on study selection, data extraction, and analyses were made in advance of the searches being conducted. The risk of bias assessment was conducted by two reviewers separately and the assessment was incorporated into the interpretation of the quality of evidence. We ensured maximum transparency regarding the methods, and therefore, this review is thought to be reproducible. The review included RCTs and, thus, regarded to have the highest level of evidence [35].

However, we recognise a few limitations of this review. First, we included the most relevant and commonly used outcomes; however, there may be other less commonly used functional outcome measures that warrant investigation. Second, we excluded observational studies and this drawback is more pertinent to the context of adverse effects of eccentric exercises like muscle soreness or injuries. RCTs are often conducted with high safety procedures, limited sample sizes, and too short follow-up times that do not allow optimal reporting of adverse effects [36]. The selection of RCTs was limited to those reporting the selected functional outcome measures and there might be studies exclusively measuring the adverse effects which have not been picked up by this review. Finally, there was vast heterogeneity in data measurement methods. This limits cross-comparison and makes it difficult to draw conclusions.

It remains important to evaluate the period for which the effects of eccentric exercise are endured in the older adult population. More importantly, it is necessary to assess the extent of reversal of training effects i.e., de-training after cessation of eccentric exercise in the older adult population, and to draw attention to the consequences of non-adherence to the exercise protocols. Qualitative studies exploring the experiences of older adults after participation in eccentric strengthening programmes are likely to add value to evidence and supplement findings from RCTs.

## Conclusions

The findings of our review suggest that eccentric-biased exercises exhibit significant improvements in balance, mobility, and endurance in healthy older adults. Furthermore, hardly any significant differences were observed, when the magnitude of these improvements was compared to those in response to concentric exercises in this population. However, the reduction in incidence of falls was greater in response to concentric exercise than to eccentric exercise. However, data on the falls’ incidence were limited and reported only in one study.

None of the studies included in this systematic review explicitly reported any episodes of injuries or undesirable outcomes. Further research is desired as explicit findings in this context were not necessarily available.

This evidence generated by this systematic review is limited by the heterogeneity across studies and small-effect sizes. Healthy older adults comprised the population of this systematic review and, therefore, there is uncertainty if the effect of eccentric exercise would be similar in older adults with any underlying health conditions.

## Supplementary Information

Below is the link to the electronic supplementary material.

Supplementary file1 (DOCX 23 kb)

## Data Availability

Not applicable. We conducted a systematic review of published literature. Search terms and databases included in the appendices.
