# Supplementary material for: Effectiveness of eccentric-biased exercise interventions in reducing the incidence of falls and improving functional performance in older adults: a systematic review
Source: Eur Geriatr Med. 2021 Oct 11;13(2):367–80. doi: 10.1007/s41999-021-00571-8 (PMC8503729; doi:10.1007/s41999-021-00571-8)
Supplement: Supplementary file 1 — Supplementary file1 (DOCX 23 kb) [file 41999_2021_571_MOESM1_ESM.docx]

*Appendix*

*Full Search strategy*

*CINAHL Plus (Ebscohost):* 18 December 2020

| S1 | (MH "Frail Elderly") OR (MH "Aged") OR (MH "Health Services for the Aged") OR (MH "Older Adult Care (Saba CCC)") OR (MH "Rehabilitation, Geriatric") OR (MH "Aged, 80 and Over") OR (MH "Geriatrics") OR "( geriatrics or older adults or elderly or aged or older or elder or elderly ) OR geriatric care OR geriatric" |
| --- | --- |
| S2 | TX eccentric exercise OR eccentric Nx2 training OR eccentric Nx2 contraction OR concentric Nx3 eccentric OR isotonic Nx5 eccentric |
| S3 | S1 AND S2 |
| S4 | TX ((balance) OR (falls OR falling)) OR ((((berg balance scale or BBS or falls efficacy scale or timed up) and go) or TUG or stair climb or stair ascen* or stair descen* or maximal walk* speed or minute walking distance or minute walking speed or chair stand)) |
| S4 | S3 AND S4 |
| Final hits: 40 | |

*Cochrane Central Register of Controlled Trials (CENTRAL, The Cochrane Library, latest issue):* 18 December 2020

| #1 | MeSH descriptor: [Aged] explode all trees |
| --- | --- |
| #2 | MeSH descriptor: [Frail Elderly] explode all trees |
| #3 | MeSH descriptor: [Health Services for the Aged] explode all trees |
| #4 | MeSH descriptor: [Geriatric Assessment] explode all trees |
| #5 | MeSH descriptor: [Geriatric Nursing] explode all trees |
| #6 | geriatric OR elderly OR old OR older OR aged OR elder |
| #7 | #1 OR #2 OR #3 OR #4 OR #5 OR #6 |
| #8 | eccentric exercise OR eccentric near/2 training OR eccentric near/2 contraction OR concentric near/3 eccentric OR isotonic near/5 eccentric |
| #9 | #7 AND #8 |
| #10 | ((balance) OR (falls OR falling)) OR ((((berg balance scale or BBS or falls efficacy scale or timed up) and go) or TUG or stair climb or stair ascen* or stair descen* or maximal walk* speed or minute walking distance or minute walking speed OR 6MWT OR chair stand)) (Word variations have been searched) |
| #11 | #9 AND #10 |
| Final hits: 113= 93(reviews)+ 19 (trials)+ 1(clinical answers) | |

*Embase (Ovid) 1980 to 2020 Week 32***:** 18 December 2020

| 1 | exp geriatric rehabilitation/ or exp geriatrician/ or geriatrics/ |
| --- | --- |
| 2 | geriatric.mp. |
| 3 | exp elderly care/ or elder$.mp. or exp aged/ |
| 4 | old$.mp. |
| 5 | 1 or 2 or 3 or 4 |
| 6 | ((((((((eccentric exercise or eccentric) adj2 training) or eccentric) adj2 contraction) or concentric) adj3 eccentric) or isotonic) adj5 eccentric).mp. [mp=title, abstract, heading word, drug trade name, original title, device manufacturer, drug manufacturer, device trade name, keyword, floating subheading word, candidate term word] |
| 7 | 5 and 6 |
| 8 | (((berg balance scale or BBS or falls efficacy scale or timed up) and go) or TUG or stair climb or stair ascen* or stair descen* or maximal walk* speed or minute walking distance or minute walking speed or chair stand).mp. [mp=title, abstract, heading word, drug trade name, original title, device manufacturer, drug manufacturer, device trade name, keyword, floating subheading word, candidate term word] |
| 9 | postural balance.mp. or exp body equilibrium/ |
| 10 | fall$.mp. or exp falling/ |
| 11 | 8 or 9 or 10 |
| 12 | 7 and 11 |
| Final hits: 83 | |

*Ovid MEDLINE(R) 1946 to July Week 5 2020:* 18 December 2020

| 1 | exp geriatric rehabilitation/ or exp geriatrician/ or geriatrics/ |
| --- | --- |
| 2 | geriatric.mp. |
| 3 | exp elderly care/ or elder$.mp. or exp aged/ |
| 4 | old$.mp. |
| 5 | 1 or 2 or 3 or 4 |
| 6 | ((((((((eccentric exercise or eccentric) adj2 training) or eccentric) adj2 contraction) or concentric) adj3 eccentric) or isotonic) adj5 eccentric).mp. [mp=title, abstract, heading word, drug trade name, original title, device manufacturer, drug manufacturer, device trade name, keyword, floating subheading word, candidate term word] |
| 7 | 5 and 6 |
| 8 | (((berg balance scale or BBS or falls efficacy scale or timed up) and go) or TUG or stair climb or stair ascen* or stair descen* or maximal walk* speed or minute walking distance or minute walking speed or chair stand).mp. [mp=title, abstract, heading word, drug trade name, original title, device manufacturer, drug manufacturer, device trade name, keyword, floating subheading word, candidate term word] |
| 9 | postural balance.mp. or exp body equilibrium/ |
| 10 | fall$.mp. or exp falling/ |
| 11 | 8 or 9 or 10 |
| 12 | 7 and 11 |
| Final hits: 44 | |

*Global Health (Ovid):* 18 December 2020

| 1 | exp geriatric rehabilitation/ or exp geriatrician/ or geriatrics/ |
| --- | --- |
| 2 | geriatric.mp. |
| 3 | exp elderly care/ or elder$.mp. or exp aged/ |
| 4 | old$.mp. |
| 5 | 1 or 2 or 3 or 4 |
| 6 | ((((((((eccentric exercise or eccentric) adj2 training) or eccentric) adj2 contraction) or concentric) adj3 eccentric) or isotonic) adj5 eccentric).mp. [mp=title, abstract, heading word, drug trade name, original title, device manufacturer, drug manufacturer, device trade name, keyword, floating subheading word, candidate term word] |
| 7 | 5 and 6 |
| 8 | (((berg balance scale or BBS or falls efficacy scale or timed up) and go) or TUG or stair climb or stair ascen* or stair descen* or maximal walk* speed or minute walking distance or minute walking speed or chair stand).mp. [mp=title, abstract, heading word, drug trade name, original title, device manufacturer, drug manufacturer, device trade name, keyword, floating subheading word, candidate term word] |
| 9 | postural balance.mp. or exp body equilibrium/ |
| 10 | fall$.mp. or exp falling/ |
| 11 | 8 or 9 or 10 |
| 12 | 7 and 11 |
| Final hits: 0 | |

| *Table 5:* *List of excluded studies with justification for exclusion at the full-text review stage* | | | |
| --- | --- | --- | --- |
|  | *First author (year)* | *Study title* | *Reason for exclusion* |
| 1. | - | Multimodal Exercise for Functional Independence in Older Women: a randomized, sham-exercise controlled clinical Trial | Could not find published results of the trial |
| 2. | Caserotti (2008) [37] | Changes in power and force generation during coupled eccentric-concentric versus concentric muscle contraction with training and aging | No randomisation: "From the eligible 161 male subjects, 44 subjects were randomly selected and divided for the present study into a training group (TG,n=16) and a control group (CG,n=28) group" |
| 3. | Chen (2017) [38] | Effects of Descending Stair Walking on Health and Fitness of Elderly Obese Women | Quasi-randomisation |
| 4. | Chen (2017) [39] | Superior effects of eccentric to concentric knee extensor resistance training on physical fitness, insulin sensitivity and lipid profiles of elderly men | Quasi-randomisation |
| 5. | Dela (2018) [40] | Eccentric versus concentric training for increases in muscle mass and strength? | Only available as a conference abstract |
| 6. | Dela (2019) [41] | Eccentric training is superior to concentric training to increase muscle mass and strength in 65 1 year healthy subjects | Only available as a conference abstract |
| 7. | - | Effects of virtual reality based exercise on mental and physical health of elderly resident in long-term care facility | Incomplete trial- ongoing recruitment |
| 8. | Hill (2020) [21] | Delayed Impairment of Postural, Physical, and Muscular Functions Following Downhill Compared to Level Walking in Older People | Single session intervention with extremely short-term outcomes |
| 9. | Hruda (2003) [33] | Training for muscle power in older adults: effects on functional abilities | Wrong intervention |
| 10. | Kochersberger (1994) [42] | Geriatrics. Do changes in strength improve balance and function in elderly men and women? | Could not retrieve the full text |
| 11. | Kubota (2019) [43] | Robot-Assisted Eccentric Contraction Training of the Tibialis Anterior Muscle Based on Position and Force Sensing | Cross-over trial |
| 12. | LaStayo (2003) [44] | The positive effects of negative work: increased muscle strength and decreased fall risk in a frail elderly population | Wrong population |
| 13. | T. J. Leszczak (2013) [45] | Early adaptations to eccentric and high-velocity training on strength and functional performance in community-dwelling older adults | Wrong intervention |
| 14. | - | Effects of Eccentric Training Intervention in Older Adults | Incomplete trial- ongoing recruitment |
| 15. | Onambélé (2008) [46] | Neuromuscular and balance responses to flywheel inertial versus weight training in older persons | Wrong intervention |
| 16. | Orr (2006) [47] | Power training improves balance in healthy older adults | Wrong outcomes |
| 17. | Raimundo (2009) [48] | Fitness efficacy of vibratory exercise compared to walking in postmenopausal women | Wrong intervention |
| 18. | Selva Raj (2011) [49] | The effects of eccentrically biased versus conventional resistance training in older adults | Only available as a conference abstract |
| 19. | Tracy (2006) [50] | Steadiness training with light loads in the knee extensors of elderly adults | Wrong intervention |

| *Table 6: Method of outcome measurement of outcomes in individual studies* | | |
| --- | --- | --- |
| *Author* | *Method of measurement of TUG* | *Unit of measurement* |
| Dias *et al.* [22] | The time taken to rise without using hands from a seated position in a chair of 43cm height, with back support, travel 2.43m, turn around a cone positioned at the end of the route, return, and sit down again in the chair (leaning back and not using hands as support). | Measured as time (s). |
| Gault *et al.* [23] | The time taken to rise from a firm, padded, armless chair, with the seat 45cm from the ground with arms folded across the chest during the sit-to-stand movements, walk 3m, turn, and return to the seated position. | Measured as time (s). |
| Johnson *et al.* [24] | The time taken to rise from a chair (of unspecified height) from a seated position, walk 3m, turn 180^0^around a cone, walk back to the chair, and return to the seated position in a controlled manner. | Measured as time (s). |
| Katsura *et al.* [25] | The time taken to stand up from a chair (of unspecified height), walk 3m, walk back to the chair and sit down. | Measured as time (s). |
| Mueller *et al.* [27] | Unspecified. | Measured as time (s). |
| Sanudo *et al*. (2019) [29] | The time taken to stand from a chair (43cm of height) without using the hands, travel 2.44m, turn around a cone positioned at the end of the route, return, and sit down in the chair again. | Measured as time (s). |
| Raj *et al.* [28] | The time taken for an individual to rise from a chair, walk 3m to touch a marker on a wall, turn 180^0^, return to the chair, and sit down. | Measured as time (s). |
| *Authors* | *Method of measurement of SCT* | *Unit of measurement* |
| Dias *et al.* [22] | Time taken to climb (unspecified speed) 8 steps of 17cm height and 31cm length, requiring a step-by-step pattern. The timer was activated when the first contact was made at the first step and ended when the contact occurred with the last step. | Measured as time (s). |
| Symons *et al.*[30] | Time taken to perform 20 two-step cycles (step-step-up, step-step-down) at own natural pace on a standard two step ergometer (20cm high x 30cm deep x 60cm wide). | Measured as time (s). |
| *Authors* | *Method of measurement of MWS* | *Unit of measurement* |
| Dias *et al*. [22] | Time taken to walk 6m at full speed with additional 3m zones marked as -3 and 9 on the ground on either side. Time was started at point 0 and ended at point 6. | Measured as time (s). |
| Gault *et al.* [23] | Time taken to walk as fast as possible along a 10m hard non-slip walkway without compromising safety. There were additional 3m zones represented by markers on either side for acceleration and deceleration. | Measured as speed (m/s). |
| Sanudo *et al.* (2020) [14] | Time taken to walk as fast as possible for 5m in a quiet corridor, without the use of any aids, wearing comfortable shoes. | Measured as speed (m/s). |
| Raj *et al.* | Time taken to walk as quickly as possible between two sets of light gates to cover 6m. | Measured as time (s). |
| *Authors* | *Method of measurement of CST* | *Unit of measurement* |
| Dias *et al.* [22] | Time taken to perform 5 repetitions of getting up and sitting down on a chair of height 43cm and a flat seat such that arms crossed over the chest throughout, raised until full extension can be observed at the trunk and lower limb joints, and return with back fully supported at the back of the chair. | Measured as time (s). |
| Gault *et al.* [23] | Time taken to stand up and sit down as fast as possible 5 times with arms folded across their chest throughout the movements on a firm, padded, armless chair, with a seat height 45cm from the ground. Time was taken from first standing up to last sitting down. | Measured as time (s). |
| Johnson *et al.* [24] | The number of sit-to-stand repetitions performed in a chair of height 44cm within 30 s by demonstrating control throughout the whole movement and to fully extend, but not lock, the hip and the knee joints upon standing. | Measured as the number of repetitions. |
| Katsura *et al.* [25] | The number of sit-to-stand repetitions performed by sitting in the middle of a chair with each hand being placed on the opposite shoulder and 2 arms crossed at the chest within 30 s. | Measured as the number of repetitions. |
| Sanudo *et al.* (2020) [14] | The number of repetitions of sit-to-stand from a chair (with a seat height of 40cm), starting with the back in an upright position and the arms folded across the chest, as many times as possible within 30 s. | Measured as number of repetitions |

37. Caserotti, P., Aagaard, P., & Puggaard, L. (2008). Changes in power and force generation during coupled eccentric-concentric versus concentric muscle contraction with training and aging. [Comparative Study; Controlled Clinical Trial; Journal Article]. *European journal of applied physiology, 103*(2), 151‐161, doi:10.1007/s00421-008-0678-x.

38. Chen, T., Chung-Chan, H., Kuo-Wei, T., Chih-Chiao, H., & Kazunori, N. (2017). Effects of Descending Stair Walking on Health and Fitness of Elderly Obese Women. *Medicine & Science in Sports & Exercise, 49*(8), 1614-1622, doi:10.1249/MSS.0000000000001267.

39. Chen, T., Tseng, W.-C., Huang, G.-L., Chen, H.-L., Tseng, K.-W., & Nosaka, K. (2017). Superior effects of eccentric to concentric knee extensor resistance training on physical fitness, insulin sensitivity and lipid profiles of elderly men. *Frontiers in physiology, 8*, 209.

40. Dela, F., Lindskov, F. O., Knudsen, A. K., Regnersgaard, S., & Pressel, E. (2018). Eccentric versus concentric training for increases in muscle mass and strength? *European Geriatric Medicine, 9 (Supplement 1)*, S95.

41. Dela, F., Mrantinkovic, M., Lindskov, F. O., Knudsen, A. K., Regnersgaard, S., & Pressel, E. (2019). Eccentric training is superior to concentric training to increase muscle mass and strength in 65 1 year healthy subjects. [Journal: Conference Abstract]. *European Geriatric Medicine, 10*, S280‐S281, doi:10.1007/s41999-019-00221-0.

42. Kochersberger, G., Studenski, S., Duncan, P., Chandler, J., & Schenckman, M. (1994). Geriatrics. Do changes in strength improve balance and function in elderly men and women? *Rehabilitation R&D Progress Reports, 30-31*, 103-104.

43. Kubota, K., Sekiya, M., & Tsuji, T. (2019). Robot-Assisted Eccentric Contraction Training of the Tibialis Anterior Muscle Based on Position and Force Sensing. *Sensors, 19*(6), 14.

44. LaStayo, P. C., Ewy, G. A., Pierotti, D. D., Johns, R. K., & Lindstedt, S. (2003). The positive effects of negative work: increased muscle strength and decreased fall risk in a frail elderly population. *Journals of Gerontology Series A: Biological Sciences & Medical Sciences, 58*(5), M419-M424, doi:10.1093/gerona/58.5.m419.

45. Leszczak, T. J., Olson, J. M., Stafford, J., & Brezzo, R. D. (2013). Early adaptations to eccentric and high-velocity training on strength and functional performance in community-dwelling older adults. [Journal Article; Randomized Controlled Trial]. *Journal of strength and conditioning research, 27*(2), 442‐448, doi:10.1519/JSC.0b013e31825423c6.

46. Onambélé, G. L., Maganaris, C. N., Mian, O. S., Tam, E., Rejc, E., McEwan, I. M., et al. (2008). Neuromuscular and balance responses to flywheel inertial versus weight training in older persons. *Journal of biomechanics, 41*(15), 3133-3138.

47. Orr, R., de Vos, N. J., Singh, N. A., Ross, D. A., Stavrinos, T. M., & Fiatarone-Singh, M. A. (2006). Power Training Improves Balance in Healthy Older Adults. *The Journals of Gerontology: Series A, 61*(1), 78-85, doi:10.1093/gerona/61.1.78.

48. Raimundo, A. M., Gusi, N., & Tomas-Carus, P. (2009). Fitness efficacy of vibratory exercise compared to walking in postmenopausal women. [Randomized Controlled Trial Research Support, Non-U.S. Gov't]. *European journal of applied physiology, 106*(5), 741-748.

49. Selva Raj, I., Bird, S., Westfold, B., & Shield, A. (2011). The effects of eccentrically biased versus conventional resistance training in older adults. *Journal of Science and Medicine in Sport, 14*, e22-e23.

50. Tracy, B. L., & Enoka, R. M. (2006). Steadiness training with light loads in the knee extensors of elderly adults. [Randomized Controlled Trial Research Support, N.I.H., Extramural]. *Medicine & Science in Sports & Exercise, 38*(4), 735-745.
